# COVID-19 Pandemic and Remote Education Contributes to Improved Nutritional Behaviors and Increased Screen Time in a Polish Population-Based Sample of Primary School Adolescents: Diet and Activity of Youth during COVID-19 (DAY-19) Study

**DOI:** 10.3390/nu13051596

**Published:** 2021-05-11

**Authors:** Aleksandra Kołota, Dominika Głąbska

**Affiliations:** Department of Dietetics, Institute of Human Nutrition Sciences, Warsaw University of Life Sciences (SGGW-WULS), 159c Nowoursynowska Street, 02-776 Warsaw, Poland; dominika_glabska@sggw.edu.pl

**Keywords:** diet, nutrition, physical activity, lifestyle, children, adolescents, primary school, COVID-19 pandemic, DAY-19 study

## Abstract

The Coronavirus-19 disease (COVID-19) pandemic has influenced the nutrition of individuals, including the diet followed, food availability, and food security. However, thus far, only a few studies have been published regarding the diet and activity of children and adolescents. The aim of the present study was to analyze the influence of the COVID-19 pandemic and remote education in this period on the diet and physical activity in a Polish population-based sample of primary school adolescents. In June 2020, the Diet and Activity of Youth during COVID-19 (DAY-19) Study was conducted on a population recruited based on stratified random sampling from all regions (schools sampled from counties, and counties from voivodeships). The sample consisted of a total of 1334 adolescents aged 10–16 years. The study assessed the diet and physical activity of the participants using a validated questionnaire which included questions about the period of remote education and the period before the COVID-19 pandemic. The participants were asked about the following: consumption of fruit, vegetables, soft drinks, water, French fries, and fast food; eating meals in front of the television; and the number of days they are physically active and the number of hours they spend watching television. The obtained data were analyzed by stratifying the respondents by the gender, age, size of the city and total COVID-19 morbidity in the voivodeship. It was observed that, during the pandemic and the resultant remote education, the proportion of respondents who declared the recommended intake of fruits and vegetables had increased compared to that before the pandemic—a higher proportion consumed at least three portions of fruit per day (19.0% before pandemic vs. 27.4% during pandemic; *p* < 0.0001), as well as three and four or more portions of vegetables per day (11.9% vs. 14.5% and 7.5% vs. 11.1%; *p* = 0.0004). At the same time, the proportion of respondents consuming at least three cups of water per day had increased (41.1% vs. 47.9%; *p* = 0.0020), whereas the proportion of respondents who never or rarely eat their meals in front of the television had decreased (35.6% vs. 28.9%; *p* < 0.0001), and the proportion watching television for more than 2 h a day had increased (78.3% vs. 88.4%; *p* < 0.0001). Based on the results, it may be concluded that, during the period of remote education due to the COVID-19 pandemic, the dietary behaviors of the studied population of Polish adolescents were more beneficial, which included a higher intake of fruit, vegetables, and water, compared to before the pandemic. In spite of the increasing screen time, including eating in front of the television, there was no reduction in the number of days the respondents were physically active.

## 1. Introduction

Coronavirus-19 disease (COVID-19), caused by severe acute respiratory syndrome coronavirus 2 (SARS-CoV-2), is a novel problem to public health and was declared as a global pandemic by the World Health Organization (WHO) on 11 March 2020 [1]. Children and adolescents affected by COVID-19 demonstrate typically a mild form of the disease [2], with different disease symptoms and clinical manifestations when compared with adults [3]. Although they exhibit milder disease, the number of adults and children who are infected has been constantly increasing [4]. So far, young infants and children with preexisting illnesses have been observed to be associated with a serious prognosis; therefore, they form a high-risk group and need careful monitoring [5].

The COVID-19 pandemic has influenced all the aspects of everyday life [6]. Among them, nutrition plays an important role at the global, national, and individual levels, including the diet followed by people, food availability, and security [7]. The pandemic has also influenced the food-buying behaviors [8], dietary habits [9], and physical activity [10] of people around the globe. The aforementioned changes have increased the risk of excessive weight gain together with quarantine-related stress and lifestyle changes [11].

The number of studies conducted on the diet and physical activity of children and adolescents is limited, and only a few have been published to date. In the study by Moore et al. [12], conducted on a group of 1472 Canadian parents of children and adolescents, the participants declared that during the COVID-19 lockdown, their children had lower levels of physical activity, decreased outdoor time, increased sedentary behaviors (including leisure screen time), and increased sleep duration. Similarly, in the study by Xiang et al. [13], conducted on a group of 2426 Chinese children and adolescents, the respondents indicated that during the COVID-19 public health emergency, there was a decrease in their physical activity but an increase in screen time. Moreover, the nationwide COVID-19 Impact on Lifestyle Change Survey (COINLICS), conducted by Jia et al. [14], indicated that during the COVID-19 lockdown, the frequency of consumption of rice, meat, poultry, fresh vegetable, fresh fruit, soybean products, and dairy products significantly decreased among youth participants. In turn, in a group of Italian obese children and adolescents, Pietrobelli et al. [15] observed that during the COVID-19 lockdown, the consumption of fruit, chips, red meat, and sugar-based drink increased, as well as sleep time and screen time, whereas the time spent in sports decreased. The study conducted by Baysun and Akar [16], on a group of Turkish toddlers, revealed that the body mass of toddlers increased, possibly due to reduced physical activity and increased food intake. A Polish study conducted on a population of 2448 secondary school adolescents, who were aged 15–20 years, showed that the pandemic changed their food choice determinants, as it increased the importance of health and weight control and reduced the role of mood and sensory appeal [17], while it also confirmed the associations between appetitive traits in the studied group [18]. This may be explained by the results of the study by Jansen et al. [19], conducted among children and adolescents aged 2–12 years, which indicated that higher COVID-19-specific stress causes a more non-nutritive intake of food and snacks, as well as more structure and positive interactions (e.g., eating with or engaging with child around mealtimes).

In studies conducted on the groups of adults in Poland, it was observed that the nutritional behaviors and physical activity of individuals have changed during the COVID-19 pandemic. In the study by Sidor and Rzymski [20], almost 50% of respondents declared increased food consumption. Similarly, the study by Błaszczyk-Bębenek et al. [21] showed increased snacking and consumption of sweets among people. Kowalczuk and Gębski [22] indicated that during the COVID-19 pandemic the respondents present changes in food purchasing, eating habits (including meal regularity and quality), and consumption of food products. At the same time, Drywień et al. [23] and Górnicka et al. [24] observed reduced levels of physical activity and increased screen time among participants.

However, the indicated nutritional and lifestyle changes are especially significant in the case of children and adolescents, as the WHO defines them as crucial for public health [25,26]. In Poland, it is stated that excessive actions are required to correct the existing problems associated with an inadequate number of meals consumed per day [27], inadequate consumption of dairy products [28], as well as fruits and vegetables [29], and excessive consumption of meat and sugar [30]. Moreover, among Polish children and adolescents, improper nutritional behaviors coexist with a decreased level of physical activity [31], with the majority of adolescents showing unsatisfactory levels [32].

Taking this into account, the present study aimed to analyze the influence of the COVID-19 pandemic and remote education in this period on the diet and physical activity in a Polish population-based sample of primary school adolescents.

## 2. Materials and Methods

### 2.1. Study Design and Studied Population

All the study procedures were approved by the Ethics Committee of the Institute of Human Nutrition Sciences of the Warsaw University of Life Sciences (no. 18/2020) and the study was conducted according to the guidelines of the Declaration of Helsinki. All participants, as well as their parents/legal guardians, provided their informed consent to participate in the study.

The Diet and Activity of Youth during COVID-19 (DAY-19) Study was conducted in June 2020, in a population-based sample of Polish primary school students. The participants were recruited based on a stratified random sampling of schools. The sampling procedure applied in this study is in agreement with those applied in other studies conducted in Poland [33]. Sampling was conducted in two phases: schools were sampled from counties, and counties were sampled from voivodeships (basic administrative unit in Poland). This sampling procedure allowed obtaining a sample that is representative of all the regions of Poland. In the first phase, the stratified sampling of schools was conducted for all regions, that is, from each voivodeship of the country (16 voivodeships), based on which 10 counties were randomly sampled (resulting in 160 counties). Later, in the second phase, 10 primary schools were randomly sampled from each county (resulting in 1600 primary schools).

All the 1600 primary schools were invited to participate in the study, by contacting their principals. The principals were informed about the aim and protocol of the study. The participation of schools in the study was voluntary. If the principal agreed to conduct the study in their school, the students and their parents/legal guardians were informed about the study. The participation of students in the study was also voluntary, and interested students provided their own informed consent along with the consent of their parents/legal guardians.

Finally, the study was conducted in 43 primary schools, which is a typical response rate for studies conducted during this period in Poland and is comparable with the response rate observed for the study by Skolmowska et al. [34]. Such a low response rate is explained by the other authors as associated with the fact that some school principals or teachers do not prefer any interruption in their classes or their school to participate in such studies [35]. The study was addressed only to students aged 10–16 years (based on the Polish primary school system attributed to 5th–8th grade, while assuming ±1 year for adolescents in the included grades), while the target group was defined as primary school adolescents, based on the WHO definition indicating adolescence as a period between 10 and 19 years [36].

**Figure 1:**
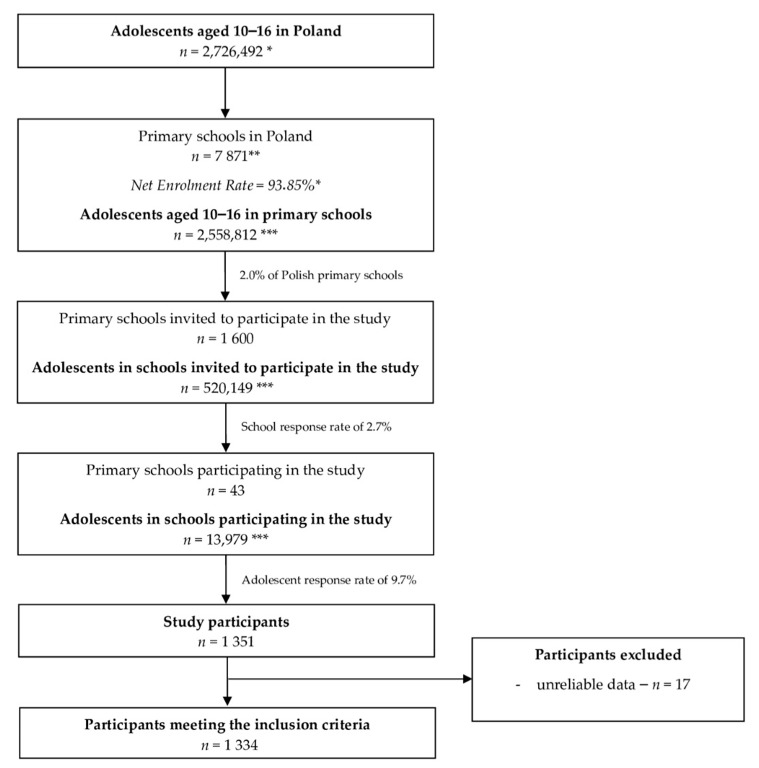
The flow chart of the sampling procedure within the Diet and Activity of Youth during COVID-19 (DAY-19) Study. * data of the Central Statistical Office (CSO) [37,38]; ** data of the Polish Ministry of National Education [39]; *** calculated based on CSO data.

The studied group of adolescents was recruited based on the response of their school principal to the invitation for participation in the study. The inclusion criteria were as follows: students of sampled schools, aged 10–16, providing informed consent of themselves as well as that of parents/legal guardians to participate in the study. The students participating in the study received an individually generated electronic link to the study questionnaire. The questionnaire was anonymous, but the respondents were asked for basic information to verify if the inclusion criteria were met. The only exclusion criterion was providing a questionnaire with unreliable data (1.26%). Finally, the sample had a total of 1334 adolescent participants (Figure 1).

### 2.2. Applied Questionnaire

In Poland, as decided by the Polish Ministry of National Education [40], primary school education has been conducted in a remote model since 12 March 2020, and remote education was still continuing when the study was conducted (June 2020). In this period, it was recommended in the country that the citizens should reduce personal contact and not leave their households unless necessary. On 16 April 2020, an additional precept was announced that the citizens should wear a face mask when leaving their households, as having the nose and mouth covered in public places was obligatory [41].

Taking this into account, the study utilized the Computer-Assisted Personal Interview (CAPI) method. The respondents were asked about their diet and physical activity for two periods—during the period of remote education due to the COVID-19 pandemic and before the COVID-19 pandemic, and a comparison of the results between these periods was made in the study. The original wording of the questions was not changed, but the respondents were asked the specific questions twice. To not refer to the general period of the COVID-19 pandemic and to enable students to understand, the questions were formulated for the period of their remote education (since 12 March 2020 [40]) and for the period before remote education (until 12 March 2020).

The applied questionnaire was developed based on the recommendations of the Sax Institute elaborated for the Ministry of Health, associated with evidence on existing validated short-form survey instruments for children’s diet, physical activity, and sedentary behavior [42]. The validated questions applied in the study are recommended to be used for adolescents aged 10 years old or over. The respondents were asked separately about their diet and physical activity, and the questionnaires were translated into Polish according to the WHO recommendations [43]. The accuracy of the translation was verified by forward translation of the questionnaires into Polish and backward translation. Additionally, the final Polish version was discussed with the group of Polish adolescents (aged 10–16) to check if the questions were understandable to them.

The study was conducted in the period of the COVID-19 pandemic. According to the general guidelines by Cade et al. [44], it is necessary to validate each questionnaire in a specific demographic group, but it is not indicated that it should be validated in a novel period. Moreover, even the WHO recommendations for questionnaire studies to be conducted within the COVID-19 period indicate that some questionnaires validated before the COVID-19 period need not be validated again in this period [45]. Taking this into account, it was assumed that there was no need to validate the questionnaire once again and the questionnaire may be considered valid even for the period of the COVID-19 pandemic.

To validate the questionnaire associated with dietary behaviors, the standardized factor loadings within the confirmatory factor analysis (CFA) were analyzed for internal reliability. The following model fit indices were calculated: Comparative Fit Index (CFI) and Root Mean Square Error of Approximation (RMSEA). A cutoff value of 0.90 or more was applied for CFI [46], and 0.06 or less for RMSEA (good fit) [47]. The CFI of 0.989 and RMSEA of 0.032 (90% CI: 0.013–0.051) observed in the study indicated a good model fit.

An additional pilot study was conducted to ensure if adolescents aged 10–16 understand the questions asked and are able to distinguish the period during and before the COVID-19 pandemic. This pilot study was conducted in a group of 12 adolescents aged 10–16, who were living in Warsaw and recruited from a primary school. It revealed that adolescents aged 10–16 are able to understand the questions asked in the questionnaire and complete the form based on their own observations of their diet and physical activity.

The questionnaire about diet was developed by Flood et al. [42] as a short form and included questions on the following: consumption of fruit, vegetables, soft drinks, water, French fried potatoes, and fast food; and eating meals in front of the television. As indicated by the Food and Agriculture Organization of the United Nations (FAO) [48], in some cases comprehensive nutrition assessment questionnaires are not practical and brief questionnaires should be applied as screeners or short dietary instruments when they are used in situations when a comprehensive assessment is not needed.

The respondents were asked about the number of portions of fruit they usually eat each day—one portion was defined as one medium piece or two small pieces of fruit or a cup of diced pieces, and the fruit group included all fresh, dried, frozen, and tinned fruit. The responses of the adolescents were clustered based on the general recommendation that at least five portions of fruits and vegetables a day should be divided into at least two portions of fruits and at least three portions of vegetables [49], as indicated in the national guidelines of many countries [50,51,52]. Accordingly, the responses were grouped into the following clusters: one portion or less, two, and three portions or more.

The respondents were asked about the number of portions of vegetables they usually eat each day—one portion was defined as half a cup of cooked vegetables or one cup of salad, and the vegetable group included all fresh, dried, frozen, and tinned vegetables. Similar to the responses obtained for fruit consumption, the responses for vegetable consumption were clustered, based on the general recommendation that at least five portions of fruits and vegetables a day should be divided into at least two portions of fruit and at least three portions of vegetables [49], as indicated in the guidelines of many countries [50,51,52]. Finally, the responses were grouped into the following clusters: two portions or less, three, and four portions or more.

The respondents were asked about the number of cups of soft drinks or sport drinks they usually drink each day/week—one can of soft drink or sport drink was assumed to correspond to 1.5 cups. The responses were clustered, as in other studies [53], into the following clusters: one cup a week or less, 2–6 cups a week, and one cup or more a day.

The respondents were asked about the number of cups of water they usually drink each day—one average bottle was attributed to two cups, and this includes tap water and bottled water. The responses were clustered, as in other studies [54], into the following clusters: less than one cup, 1–2 cups, and two cups or more.

The respondents were asked about the frequency of consumption of hot chips, wedges, or hot French fried potatoes. The responses were clustered, as in other studies [55] into the following clusters: less than once a week, 1–2 times a week, and two or more times a week.

The respondents were asked about the frequency of consumption of fast food, including meals or snacks, such as burgers, pizza, chicken, or chips from takeaway food places. The responses were clustered, as in other studies [55], into the following clusters: less than once a week, 1–2 times a week, and two or more times a week.

The respondents were asked about the frequency of eating meals in front of the television. The responses were clustered, as in other studies [56], into the following clusters: never or rarely, 1–4 times a week, 5–6 times a week, and every day.

The questionnaire about physical activity, as described by Flood et al. [42], was developed and validated by Prochaska et al. [57]. It included questions on the following: number of days a week the respondents are physically active and number of hours a day the respondents usually spend watching television. Similar to diet evaluation, the brief-form questionnaire was applied for the analysis of the physical activity of the respondents.

The respondents were asked about the number of days a week they are physically active for a total of at least 60 min per day and were asked to include all the time except for their physical education or gym class. While asked about their physical activity, the respondents were informed to include any activity that increases their heart rate and makes them get out of breath some of the time, during their physical education or gym class (e.g., running, biking, dancing, skateboarding, swimming, soccer). Based on the WHO recommendations for children that vigorous-intensity activities should be incorporated at least three times per week [58], the responses of the respondents were grouped into the following clusters: two days or less, and three days or more.

The respondents were asked about the number of hours a day they usually spend watching television, during their weekdays. The responses were clustered, as in other studies [59,60], into the following clusters: less than 2 h a day, and two or more hours a day. The cut-off of 2 h was assumed based on the general recommendations for children and adolescents that their television time should be no longer than 2 h per day [61]. This is also in accordance with the results of the systematic review by Tremblay et al. [62] indicating that watching television for more than 2 h daily is associated with reduced physical and psychosocial health.

### 2.3. Statistical Analysis

The sample size was calculated considering the population of Polish adolescents aged 10–16 years (2,726,492, based on the data from the Central Statistical Office (CSO) in Poland [63]), at a 95% confidence level and 5% margin of error. Assuming a percentage of 50%, the required sample size was estimated at 384 respondents. Thus, the gathered sample of 1334 respondents was interpreted as sufficient.

The respondents were stratified by gender, age, size of the city, and the total COVID-19 morbidity in the voivodeship until June 2020 (when the study was conducted). The total COVID-19 morbidity in the voivodeship was included within the other factors, as in Poland, during the period of the pandemic, it was stated to be associated with lifestyle behaviors presented by individuals [34]. Moreover, other studies indicated that there is an association between the COVID-19 mortality and dietary behaviors [64]. The total COVID-19 morbidity in the voivodeship was calculated based on the number of COVID-19 cases diagnosed in the indicated period and the number of inhabitants. Afterward, the voivodeships were divided according to the number of COVID-19 cases per 100,000 inhabitants as less than 40, 40–80, 80–160, and more than 160 cases, based on the data of the Polish Government [65]. Such an approach was in agreement with those chosen in other Polish studies [34]. This is associated with the fact that in the studied period, various regions of Poland were characterized by extremely diverse morbidity—in April 2020, 6 voivodeships had more than 70% of total COVID-19 cases in Poland, while the remaining 10 voivodeships had less than 30% of total cases [65].

To compare responses provided by adolescents for the period before and during the period of remote education due to the COVID-19 pandemic, as well as responses provided by adolescents stratified by gender, age, size of the city, and the total COVID-19 morbidity in the voivodeship, the chi2 test was used. The value of *p* ≤ 0.05 was interpreted as statistically significant. All statistical analyses were conducted while using Statgraphics Plus for Windows 4.0 (Statgraphics Technologies Inc., The Plains, VA, USA).

## 3. Results

The general characteristics of the studied group of adolescents are presented in Table 1. It should be indicated that a higher proportion of girls than boys participated in the study, while the general Polish population of adolescents in the studied age group has a higher proportion of boys than girls.

The analysis of the responses to the questions on fruit and vegetable consumption provided by the studied group is presented in Table 2. It was observed that, during the period of remote education due to the COVID-19 pandemic, the share of respondents declaring the recommended intake of fruits and vegetables was higher in comparison to before the COVID-19 pandemic, as the higher share of respondents declared consuming at least three portions of fruit a day (27.4% vs. 19.0%), as well as three (14.5% vs. 11.9%) and four or more portions of vegetables per day (11.1% vs. 7.5%). The detailed analysis of fruit and vegetable consumption declared by the studied sample of Polish adolescents, stratified by gender, age, size of the city, and COVID-19 morbidity in voivodeship, is presented in Supplementary Tables S1 and S2, respectively. The same patterns were observed in analyses stratified by gender, age, size of the city, and COVID-19 morbidity in voivodeship.

The analysis of responses to the questions on soft drinks and water consumption provided by the studied group is presented in Table 3. It was observed that, during the period of remote education due to the COVID-19 pandemic, the declared soft drinks consumption in the studied group was the same as before the COVID-19 pandemic. At the same time, the share of respondents declaring recommended water intake was higher than before the COVID-19 pandemic, as the higher share of respondents declared consuming at least three cups per day (47.9% vs. 41.1%). The detailed analysis of soft drinks and water consumption declared by the studied sample of Polish adolescents, stratified by gender, age, size of the city, and COVID-19 morbidity in voivodeship, is presented in Supplementary Tables S3 and S4, respectively. The same situation was observed in analyses stratified by gender, age, size of the city, and COVID-19 morbidity in voivodeship, as similarly, no significant associations were stated in a majority of sub-groups.

The analysis of responses to the questions on French fried potatoes and fast-food consumption provided by the studied group is presented in Table 4. It was observed that during the period of remote education due to the COVID-19 pandemic, the declared French fried potatoes and fast food consumption in the studied group was the same as before the COVID-19 pandemic. The detailed analysis of French fried potatoes and fast food consumption declared by the studied sample of Polish adolescents, stratified by gender, age, size of the city, and COVID-19 morbidity in voivodeship, is presented in Supplementary Tables S5 and S6, respectively. The same situation was observed in analyses stratified by gender, age, size of the city, and COVID-19 morbidity in voivodeship, as similarly no significant associations were stated in a majority of sub-groups.

The analysis of responses to the questions on frequency of eating their meals in front of the television, number of days a week when they are physically active, and number of hours a day that they usually spend watching television provided by the studied group is presented in Table 5. It was observed that, during the period of remote education due to the COVID-19 pandemic, the share of respondents declaring eating their meals in front of the television never or rarely was lower than before the COVID-19 pandemic (28.9% vs. 35.6%), as well as the share of respondents declaring watching television more than 2 h a day was higher than before the COVID-19 pandemic (88.4% vs. 78.3%). At the same time, the declared number of days a week when they are physically active in the studied group was the same as before the COVID-19 pandemic. The detailed analysis of eating their meals in front of the television, the number of days a week when they are physically active and the number of hours a day that they usually spend watching television declared by the studied sample of Polish adolescents, stratified by gender, age, size of the city, and COVID-19 morbidity in voivodeship, is presented in Supplementary Tables S7–S9, respectively. The same patterns were observed in analyses stratified by gender, age, size of the city, and COVID-19 morbidity in voivodeship.

## 4. Discussion

In Poland, the spread of SARS-CoV-2 has caused a number of restrictions, including the enforcement of remote education [40], in addition to limitations such as avoiding personal contact, following social distancing, and going out of home only when necessary [41]. All these regulations have influenced the lifestyle of both adults and children [24]. Reduced physical activity was observed in adults during the COVID-19 lockdown, and its consequences should be counteracted by a properly planned education [66]. Similarly, food insecurity (defined as a lack of consistent access to enough food for an active healthy life [67]) and changing dietary behaviors are challenging and may contribute to an improperly balanced diet [68].

It has been understood that the COVID-19 pandemic significantly alters all the aspects of daily life, including general health, which is influenced by changing lifestyles, lockdown, social isolation, and economic consequences of the pandemic [69]. Jribi et al. [8] reported that people were showing increasing interest in preventing food wastage, not due to the ecological awareness, but rather due to the economic burden of households resulting from reduced income and insecurity [70]. Such a situation may cause changes in consumption, not only because of the closure of restaurants or bars, but also due to the increasing prices of imported products, increasing costs of production in the country, and changes in export structure [71]. Especially regarding fruits and vegetables and other perishable food products, which may not be perceived as essential, the COVID-19 pandemic may have reduced their consumption in households [72].

It should be emphasized that the findings of the present study indicate a potentially beneficial situation, as it was observed that the respondents did not reduce their frequency of fruit and vegetable consumption and in fact increased the consumption during the period of remote education due to the pandemic. This observation corresponds with the fact that the majority of nutritional recommendations provided for the COVID-19 period encouraged the consumption of fruits and vegetables [73].

Similar results of positive diet changes during the COVID-19 pandemic have been reported by Ruiz-Roso et al. [74], who stated that patients with type 2 diabetes mellitus increased their vegetable consumption, and by Laguna et al. [75], who stated that the Spanish population increased their frequency of purchasing vegetables. This may be associated with health motivations [76], as it was also observed in a previous study of Polish adolescents, as in the period of COVID-19, health was declared as a more important food choice determinant by the participants [17]. In addition, during the lockdown, families may have more time to cook in their households [74], which might be another determinant of increased vegetable consumption during the ongoing pandemic. It is noteworthy that homemade dishes contain lesser amounts of salt, fat, and sugar compared to processed products [76]; therefore, it can be supposed that not only the intake of fruits and vegetables has increased but also the overall quality of diet has changed.

Regarding beverage consumption, some positive changes were also observed among the Polish population of primary school adolescents. They did not increase their intake of soft drinks but rather increased the consumption of water. Di Renzo et al. [69] reported similar observations in their study on a population of Italian children. Such increased water intake during the COVID-19 pandemic and lockdown may be a result of remote education—at schools, children and adolescents may have limited access to water [77], but staying at home may increase water consumption. Moreover, the constant contact with parents at home may be another reason for increased water consumption, as parents may promote positive nutritional behaviors, including water intake [78]. Thus, nutritional campaigns may be effective if they are conducted in a family setting [79].

These results are highly encouraging considering the fact that stress and depression increase the consumption of sugar-based food products, including soft drinks, by individuals for boosting their mood [80], and the COVID-19 pandemic has become a major stressor [81].

Increased water consumption is not only important for general health [82] but is also important during the COVID-19 pandemic. Experimental, clinical, and epidemiological evidence suggests that chronic suboptimal hydration before SARS-CoV-2 infection may increase the risk of mortality; therefore, public health recommendations insist that the intake of drinking water should be increased [83].

In the context of COVID-19, which may lead to prolonged stress and its related consequences, a properly balanced diet plays an important role [84]. It is known that the consumption of fruits and vegetables may have a positive effect on general mental health, as it may promote higher levels of optimism and self-efficacy, reduce psychological distress, and protect against depressive symptoms [85]. Similarly, water intake may contribute to a decreased risk of depression and anxiety [86]. Taking this into account, it can be assumed that the observed changes in dietary behaviors are beneficial, as they may promote not only the physical but also the mental well-being of individuals.

However, in addition to dietary behaviors, lifestyle changes should be analyzed in Polish adolescents. It has been indicated that lockdown and the resultant remote education may reduce the level of physical activity in children [87]. This is due to the limited possibility of having an organized physical education or gym class, as well as the restrictions of spontaneous physical activity associated with the necessities of daily out-of-home life [88]. Moreover, Xiang et al. [13] reported that a significant reduction of physical activity may affect not only physical health but also mental health.

Before the COVID-19 pandemic, poor physical activity was stated as a common problem, leading to excessive body mass in children and adolescents [89]. The ongoing pandemic has further increased the sedentary lifestyle of people [90]. In the present study, the participants were asked about the number of days they were physically active, but except for their physical education or gym class, as it is obvious that school physical education is suspended due to lockdown and remote education [91]. While asked about other active days, the participants stated no difference comparing the period before and during the pandemic. It may be due to the fact that this study was conducted in June 2020, which is one of the warmest months in Poland; therefore, the participants may have been physically active but may have chosen those physical activities that were allowed (individual sports such as riding a bike or running), instead of team sports that were restricted due to social distancing.

Similar to our study, a previous study has reported that during the COVID-19 pandemic, children and adolescents spend more time watching television [60]. Such behaviors, which are common especially among young individuals, may above all promote a sedentary lifestyle with reduced energy expenditure [92]. Moreover, depending on the type of food products consumed and the energy value of diet, sedentary lifestyle and more screen time may increase the risk of obesity [93].

The problem of insufficient physical activity may be more prominent among children with an excessive body mass. This was also indicated by Pietrobelli et al. [15], who stated that during the COVID-19 pandemic obese adolescents have spent less time being physically active, but more time watching television compared to other participants of the study, as well as declared increased consumption of sweets and snacks. Hence, the COVID-19 period may exacerbate the existing problem of excessive body mass in children and adolescents. Similar conclusions may be formulated from results of the study by Yang et al. [94], who observed that during lockdown Chinese adolescents were less engaged in active transport, household works of moderate-to-vigorous intensity, physical activity of moderate-to-vigorous intensity, and walking, but had increased leisure time, screen time, and sleeping duration.

Regarding eating in front of the television, the present study showed that this behavior was more common during the period of remote education due to COVID-19 than the period before. It should also be indicated that the main change was associated with an increase in the share of adolescents eating in front of the television 1–4 times per week (instead of doing it never or rarely, as they did before pandemic), while the share of adolescents doing it 5–6 or 7 times per week did not increase. Moreover, while this behavior was not assessed in terms of the food products consumed in front of the television, it cannot be undoubtedly interpreted as a negative behavior. Such behaviors may be natural for the period when children are forced to stay at home, as stated in the other studies that in this period both total screen time and leisure screen time increased [88].

At the same time, the negative lifestyle changes resulting from lockdown and remote learning system may be a serious problem, as it may consequently lead to increased body mass and reduced sleeping time in the case of younger children [95], and getting worse school grades in the case of older children [96]. Taking this into account, it is essential to formulate dedicated recommendations for children and adolescents, to counteract their negative lifestyle changes associated with lockdown and the resultant remote learning.

This study has both strengths and limitations. Its major strengths are the sample size and the recruitment procedure which allowed gathering respondents from all the regions of Poland. Another strength is that it used a validated questionnaire dedicated to the studied age group. Moreover, the analysis was conducted not only in the general sample, but also in the subsamples stratified by various parameters such as gender, age, size of the city, and the total COVID-19 morbidity in the voivodeship. At the same time, it should be indicated that the study had a low school response rate and a higher proportion of girls than boys participated in the study which influenced the representativeness of the studied group. In addition, the applied questionnaire included only some aspects of diet and physical activity, and so a comprehensive analysis was not possible. The other issue is the general risk of recall bias which may be associated with inaccurate and incomplete recollection of events by the respondents. Last but not least, the applied questionnaire was not validated for the COVID-19 pandemic, and hence it cannot be stated that this period does not influence the results obtained using this form.

Taking into account all the discussed differences in diet and physical activity before and during the period of remote education due to the COVID-19 pandemic, it must be emphasized that the current pandemic is probably not just a problem faced in 2020 but is a long-term issue. Until a COVID-19 vaccine becomes commonly available, the recommended restrictions should be followed. Adolescents should be made aware of the situation and helped to cope with the current situation and changing lifestyle during lockdown and remote education. Moreover, further studies should be conducted including an assessment of the influence of the COVID-19 pandemic and related lockdown or remote education on general nutritional behaviors and physical activity patterns. Such studies should be conducted in various countries or regions and in various seasons, and also assess the impact of other potential influencing factors, such as household situation, COVID-19-associated experiences of the family, or the general approach of parents or legal guardians toward COVID-19.

## 5. Conclusions

It may be concluded that the COVID-19 pandemic and remote education in this period influenced the diet and physical activity in a Polish population-based sample of primary school adolescents. More beneficial dietary behaviors were observed during the period of remote education due to the COVID-19 pandemic than before, which include a higher intake of fruit, vegetable, and water. However, increased screen time, including eating in front of the television, was also observed in comparison to the period before the COVID-19 pandemic, but there was no influence on the number of days the adolescents were physically active.

## Figures and Tables

**Table 1:** The general characteristics of the studied group of Polish adolescents within the Diet and Activity of Youth during COVID-19 (DAY-19) Study.

Parameter		Adolescents Stratified by COVID-19 Morbidity for the Voivodeship				Total
		<40/ 100,000	40–80/ 100,000	80–160/ 100,000	>160/ 100,000	
Total number of adolescents		413	537	209	175	1334
Gender	Girls	240	264	116	91	711
	Boys	173	273	93	84	623
Age (years)	10–11	42	51	13	14	120
	11–12	90	133	41	50	314
	12–13	95	125	38	36	294
	13–14	89	115	52	33	289
	14–15	75	68	42	27	212
	15–16	22	45	23	15	105

**Table 2:** Analysis of declared fruit and vegetable consumption in the studied group of Polish adolescents within the Diet and Activity of Youth during COVID-19 (DAY-19) Study.

Assessed Consumption	Portions per Day	before COVID-19	during Remote Education Due to COVID-19	*p*-Value
Fruit consumption	0–1	597 (44.7%)	485 (36.4%)	<0.0001
	2	484 (36.3%)	483 (36.2%)	
	≥3	253 (19.0%)	366 (27.4%)	
Vegetable consumption	0-2	1074 (80.5%)	992 (74.4%)	0.0004
	3	159 (11.9%)	194 (14.5%)	
	≥4	101 (7.5%)	148 (11.1%)	

**Table 3:** Analysis of declared soft drinks and water consumption in the studied group of Polish adolescents within Diet and Activity of Youth during COVID-19 (DAY-19) Study.

Assessed Consumption	Portions	before COVID-19	during Remote Education Due to COVID-19	*p*-Value
Soft drinks consumption	≥1/week	792 (59.4%)	795 (59.6%)	0.8275
	2–6/week	396 (29.7%)	385 (28.9%)	
	≥1/day	146 (10.9%)	154 (11.5%)	
Water consumption	<1/day	148 (11.1%)	137 (10.3%)	0.0020
	1–2/day	637 (47.8%)	558 (41.8%)	
	≥3/day	549 (41.1%)	639 (47.9%)	

**Table 4:** Analysis of declared French fried potatoes and fast-food consumption in the studied group of Polish adolescents within the Diet and Activity of Youth during COVID-19 (DAY-19) Study.

Assessed Consumption	Portions per Week	before COVID-19	during Remote Education Due to COVID-19	*p*-Value
French fried potatoes consumption	>1	1079 (80.9%)	1054 (79.0%)	0.3657
	1–2	213 (15.9%)	227 (17.0%)	
	≥2	42 (3.2%)	53 (4.0%)	
Fast food consumption	>1	1197 (89.7%)	1187 (88.9%)	0.4135
	1–2	113 (8.5%)	113 (8.5%)	
	≥2	24 (1.8%)	34 (2.6%)	

**Table 5:** Analysis of declared frequency of eating their meals in front of the television, number of days a week when they are physically active and number of hours a day that they usually spend watching television in the studied group of Polish adolescents within the Diet and Activity of Youth during COVID-19 (DAY-19) Study.

Category of COVID-19 Morbidity for the Voivodeship	Frequency	before COVID-19	during Remote Education Due to COVID-19	*p*-Value
Frequency of eating their meals in front of the television	Never or rarely	475 (35.6%)	385 (28.9%)	<0.0001
	1–4/week	509 (38.2%)	649 (48.6%)	
	5–6/week	156 (11.7%)	129 (9.7%)	
	7/week	194 (14.5%)	171 (12.8%)	
Number of days when they are physically active	0–2/week	542 (40.6%)	501 (37.6%)	0.1125
	≥3/week	792 (59.4%)	833 (62.4%)	
Number of hours that they spend watching television	<2 h/day	289 (21.7%)	155 (11.6%)	<0.0001
	≥2 h/day	1045 (78.3%)	1179 (88.4%)	

## Supplementary Materials

The following are available online at https://www.mdpi.com/article/10.3390/nu13051596/s1; Table S1. Analysis of declared fruit consumption in the studied group of Polish adolescents stratified by gender, age, size of the city and COVID-19 morbidity in voivodeship, Table S2. Analysis of declared vegetable consumption in the studied group of Polish adolescents stratified by gender, age, size of the city and COVID-19 morbidity in voivodeship, Table S3. Analysis of declared soft drinks consumption in the studied group of Polish adolescents stratified by gender, age, size of the city and COVID-19 morbidity in voivodeship, Table S4. Analysis of declared water consumption in the studied group of Polish adolescents stratified by gender, age, size of the city and COVID-19 morbidity in voivodeship, Table S5. Analysis of declared French fried potatoes consumption in the studied group of Polish adolescents stratified by gender, age, size of the city and COVID-19 morbidity in voivodeship, Table S6. Analysis of declared fast food consumption in the studied group of Polish adolescents stratified by gender, age, size of the city and COVID-19 morbidity in voivodeship, Table S7. Analysis of declared frequency of eating their meals in front of the television in the studied group of Polish adolescents stratified by gender, age, size of the city and COVID-19 morbidity in voivodeship, Table S8. Analysis of declared number of days a week when they are physically active in the studied group of Polish adolescents stratified by gender, age, size of the city and COVID-19 morbidity in voivodeship, Table S9. Analysis of declared number of hours a day that they usually spend watching television in the studied group of Polish adolescents stratified by gender, age, size of the city and COVID-19 morbidity in voivodeship.
