# Supplementary material for: COVID-19 Pandemic and Remote Education Contributes to Improved Nutritional Behaviors and Increased Screen Time in a Polish Population-Based Sample of Primary School Adolescents: Diet and Activity of Youth during COVID-19 (DAY-19) Study
Source: Nutrients. 2021 May 11;13(5):1596. doi: 10.3390/nu13051596 (PMC8151489; doi:10.3390/nu13051596)
Supplement: Supplementary file 1 [file nutrients-13-01596-s001.zip › nutrients-1159183-supplementary.pdf]

nutrients

MDPI

Supplementary Materials

COVID-19 Pandemic and Remote Education Contributes Improved Nutritional Behaviours and Increased Screen Time in a Polish Population-Based Sample of Primary School Adolescents: Diet and Activity of Youth during COVID-19 (DAY-19) Study

Aleksandra Kołota *, Dominika Głąbska

Table S1. Analysis of declared fruit consumption in the studied group of Polish adolescents stratified by gender, age, size of the city and COVID-19 morbidity in voivodeship.

Category	Portions per day	Before COVID-19	During remote education due to COVID-19	p-Value
**Gender**				
Girls (*n* = 711)	0-1	29 (4.1%)	30 (4.2%)	<0.0001
	2	274 (38.5%)	188 (26.5%)	
	≥3	408 (57.4%)	493 (69.3%)	
Boys (*n* = 623)	0-1	38 (6.1%)	39 (6.3%)	0.2354
	2	257 (41.2%)	228 (36.6%)	
	≥3	328 (52.7%)	356 (57.1%)	
**Age (years)**				
10-13 (*n* = 1017)	0-1	447 (43.9%)	388 (38.1%)	0.0001
	2	375 (36.9%)	352 (34.6%)	
	≥3	195 (19.2%)	277 (27.2%)	
13-16 (*n* = 317)	0-1	150 (47.3%)	97 (30.6%)	<0.0001
	2	109 (34.4%)	131 (41.3%)	
	≥3	58 (18.3%)	89 (28.1%)	
**Size of the city**				
Urban (*n* = 968)	0-1	445 (45.9%)	371 (38.3%)	<0.0001
	2	353 (36.5%)	343 (35.4%)	
	≥3	170 (17.6%)	254 (26.3%)	
Rural (*n* = 366)	0-1	152 (41.5%)	114 (31.1%)	<0.0001
	2	131 (35.8%)	112 (30.6%)	
	≥3	83 (22.7%)	140 (38.3%)	
**COVID-19 morbidity for the voivodeship**				
Morbidity < 40/ 100.000 (*n* = 413)	0-1	196 (47.2%)	143 (34.6%)	0.0004
	2	140 (33.9%)	160 (38.8%)	
	≥3	77 (18.6%)	110 (14.5%)	
Morbidity 40-80/ 100.000 (*n* = 537)	0-1	241 (44.9%)	225 (41.9%)	0.0099
	2	196 (36.5%)	171 (31.8%)	
	≥3	100 (18.6%)	141 (26.3%)	
Morbidity 80-160/ 100.000	0-1	85 (40.7%)	60 (28.7%)	0.0056

(*n* = 209)	2	77 (36.8%)	75 (35.9%)	
	≥3	47 (22.5%)	74 (35.4%)	
Morbidity >160/ 100.000 (*n* = 175)	0-1	75 (42.8%)	57 (32.6%)	0.0928
	2	71 (40.6%)	77 (44.0%)	
	≥3	29 (16.6%)	41 (23.4%)	
Total (*n* = 1334)	0-1	597 (44.7%)	485 (36.4%)	<0.0001
	2	484 (36.3%)	483 (36.2%)	
	≥3	253 (19.0%)	366 (27.4%)	

Table S2. Analysis of declared vegetable consumption in the studied group of Polish adolescents stratified by gender, age, size of the city and COVID-19 morbidity in voivodeship.

Category	Portions per day	Before COVID-19	During remote education due to COVID-19	p-Value
**Gender**				
Girls (*n* = 711)	0-2	343 (48.2%)	278 (39.1%)	0.0004
	3	236 (33.2%)	249 (35.0%)	
	≥4	132 (18.6%)	184 (25.9%)	
Boys (*n* = 623)	0-2	312 (50.1%)	286 (45.9%)	0.1152
	3	183 (29.4%)	179 (28.7%)	
	≥4	128 (20.5%)	158 (25.4%)	
**Age (years)**				
10-13 (*n* = 1017)	0-2	821 (80.7%)	744 (75.1%)	0.0044
	3	113 (11.1%)	145 (14.3%)	
	≥4	83 (8.2%)	108 (10.6%)	
13-16 (*n* = 317)	0-2	253 (79.8%)	228 (71.9%)	0.0077
	3	46 (14.5%)	49 (15.5%)	
	≥4	18 (5.7%)	40 (12.6%)	
**Size of the city**				
Urban (*n* = 968)	0-2	784 (81.0%)	715 (73.9%)	0.0007
	3	115 (11.9%)	150 (15.5%)	
	≥4	69 (7.1%)	103 (10.6%)	
Rural (*n* = 366)	0-2	290 (79.2%)	277 (75.7%)	0.2875
	3	44 (12.0%)	44 (12.0%)	
	≥4	32 (8.8%)	45 (12.3%)	
**COVID-19 morbidity for the voivodeship**				
Morbidity < 40/ 100.000 (*n* = 413)	0-2	345 (83.5%)	312 (75.6%)	0.0173
	3	43 (10.4%)	63 (15.2%)	
	≥4	25 (6.1%)	38 (9.2%)	
Morbidity 40-80/ 100.000 (*n* = 537)	0-2	434 (80.7%)	412 (76.8%)	0.2113
	3	57 (10.6%)	62 (11.5%)	
	≥4	47 (8.7%)	63 (11.7%)	
Morbidity 80-160/ 100.000 (*n* = 209)	0-2	150 (71.8%)	132 (63.2%)	0.0739
	3	38 (18.2%)	41 (19.6%)	
	≥4	21 (10.0%)	36 (17.2%)	
Morbidity >160/ 100.000 (*n* = 175)	0-2	146 (83.4%)	136 (77.7%)	0.4009
	3	21 (12.0%)	28 (16.0%)	

	≥4	8 (4.6%)	11 (6.3%)	
Total<br>(*n* = 1334)	0-2	146 (83.4%)	136 (77.7%)	0.0004
	3	21 (12.0%)	28 (16.0%)	
	≥4	8 (4.6%)	11 (6.3%)	

Table S3. Analysis of declared soft drinks consumption in the studied group of Polish adolescents stratified by gender, age, size of the city and COVID-19 morbidity in voivodeship.

Category	Portions	Before COVID-19	During remote education due to COVID-19	p-Value
**Gender**				
Girls (*n* = 711)	≥1/week	446 (62.7%)	449 (63.1%)	0.8367
	2-6/week	199 (28.0%)	191 (26.9%)	
	≥1/day	66 (9.3%)	71 (10.0%)	
Boys (*n* = 623)	≥1/week	346 (55.6%)	346 (55.6%)	0.9616
	2-6/week	197 (31.6%)	194 (31.1%)	
	≥1/day	80 (12.8%)	83 (13.3%)	
**Age (years)**				
10-13 (*n* = 1017)	≥1/week	629 (61.9%)	617 (60.7%)	0.7610
	2-6/week	295 (29.0%)	298 (29.3%)	
	≥1/day	93 (9.1%)	102 (10.0%)	
13-16 (*n* = 317)	≥1/week	163 (51.4%)	178 (56.2%)	0.4249
	2-6/week	101 (31.9%)	87 (27.4%)	
	≥1/day	53 (16.7%)	52 (16.4%)	
**Size of the city**				
Urban (*n* = 968)	≥1/week	570 (58.9%)	581 (60.0%)	0.5117
	2-6/week	293 (30.3%)	272 (28.1%)	
	≥1/day	105 (10.8%)	115 (11.9%)	
Rural (*n* = 366)	≥1/week	222 (60.4%)	214 (58.5%)	0.719
	2-6/week	103 (28.1%)	113 (30.9%)	
	≥1/day	41 (11.2%)	39 (10.6%)	
**COVID-19 morbidity for the voivodeship**				
Morbidity < 40/ 100.000 (*n* = 413)	≥1/week	248 (60.0%)	247 (59.8%)	0.9091
	2-6/week	113 (27.3%)	110 (26.6%)	
	≥1/day	52 (13.6%)	56 (13.6%)	
Morbidity 40-80/ 100.000 (*n* = 537)	≥1/week	305 (56.8%)	311 (57.9%)	0.8296
	2-6/week	181 (33.7%)	172 (32.0%)	
	≥1/day	51 (9.5%)	54 (10.1%)	
Morbidity 80-160/ 100.000 (*n* = 209)	≥1/week	121 (57.9%)	122 (58.4%)	0.9938
	2-6/week	60 (28.7%)	59 (28.2%)	
	≥1/day	28 (13.4%)	28 (13.4%)	
Morbidity >160/ 100.000 (*n* = 175)	≥1/week	118 (67.4%)	115 (65.7%)	0.9430
	2-6/week	42 (24.0%)	44 (25.2%)	
	≥1/day	15 (8.6%)	16 (9.1%)	
Total (*n* = 1334)	≥1/week	792 (59.4%)	795 (59.6%)	0.8275
	2-6/week	396 (29.7%)	385 (28.9%)	
	≥1/day	146 (10.9%)	154 (11.5%)	

Table S4. Analysis of declared water consumption in the studied group of Polish adolescents stratified by gender, age, size of the city and COVID-19 morbidity in voivodeship.

Category	Portions per day	Before COVID-19	During remote education due to COVID-19	p-Value
**Gender**				
Girls (*n* = 711)	<1	91 (12.8%)	74 (10.4%)	0.1039
	1-2	218 (30.7%)	197 (27.7%)	
	≥3	402 (56.5%)	440 (61.9%)	
Boys (*n* = 623)	<1	57 (9.1%)	63 (10.1%)	0.4321
	1-2	188 (30.2%)	168 (27.0%)	
	≥3	378 (60.6%)	392 (62.9%)	
**Age (years)**				
10-13 (*n* = 1017)	<1	107 (10.5%)	101 (9.9%)	0.4182
	1-2	310 (30.5%)	287 (28.2%)	
	≥3	600 (59.0%)	629 (61.9%)	
13-16 (*n* = 317)	<1	41 (12.9%)	36 (11.4%)	0.1680
	1-2	96 (30.3%)	78 (24.6%)	
	≥3	180 (56.8%)	203 (64.0%)	
**Size of the city**				
Urban (*n* = 968)	<1	105 (10.9%)	106 (11.0%)	0.2792
	1-2	284 (29.3%)	253 (26.1%)	
	≥3	579 (59.8%)	609 (62.9%)	
Rural (*n* = 366)	<1	43 (11.7%)	31 (8.5%)	0.2765
	1-2	112 (30.6%)	112 (30.6%)	
	≥3	211 (57.7%)	223 (60.9%)	
**COVID-19 morbidity for the voivodeship**				
Morbidity < 40/ 100.000 (*n* = 413)	<1	46 (11.1%)	37 (8.9%)	0.0199
	1-2	197 (47.7%)	166 (40.3%)	
	≥3	170 (41.2%)	210 (50.8%)	
Morbidity 40-80/ 100.000 (*n* = 537)	<1	71 (13.2%)	73 (13.6%)	0.2549
	1-2	236 (43.9%)	210 (39.1%)	
	≥3	230 (42.9%)	254 (47.3%)	
Morbidity 80-160/ 100.000 (*n* = 209)	<1	15 (7.2%)	13 (6.2%)	0.1759
	1-2	106 (50.7%)	89 (42.6%)	
	≥3	88 (42.1%)	107 (51.2%)	
Morbidity >160/ 100.000 (*n* = 175)	<1	16 (9.1%)	14 (8.0%)	0.7247
	1-2	98 (56.0%)	93 (53.1%)	
	≥3	61 (34.9%)	68 (38.9%)	
Total (*n* = 1334)	<1	148 (11.1%)	137 (10.3%)	0.0020
	1-2	637 (47.8%)	558 (41.8%)	
	≥3	549 (41.1%)	639 (47.9%)	

Table S5. Analysis of declared French fried potatoes consumption in the studied group of Polish adolescents stratified by gender, age, size of the city and COVID-19 morbidity in voivodeship.

Category	Portions per week	Before COVID-19	During remote education due to COVID-19	p-Value
**Gender**				
Girls (*n* = 711)	<1	576 (81.0%)	571 (80.3%)	0.5822
	1-2	115 (16.2%)	113 (15.9%)	
	≥2	20 (2.8%)	27 (3.8%)	
Boys (*n* = 623)	<1	503 (80.7%)	483 (77.5%)	0.3778
	1-2	98 (15.7%)	114 (18.3%)	
	≥2	22 (3.6%)	26 (4.2%)	
**Age (years)**				
10-13 (*n* = 1017)	<1	830 (81.6%)	809 (79.6%)	0.4164
	1-2	158 (15.5%)	171 (16.8%)	
	≥2	29 (2.9%)	37 (3.6%)	
13-16 (*n* = 317)	<1	249 (78.6%)	245 (77.4%)	0.8999
	1-2	55 (17.3%)	57 (17.9%)	
	≥2	13 (4.1%)	15 (4.7%)	
**Size of the city**				
Urban (*n* = 968)	<1	783 (80.9%)	766 (79.2%)	0.3957
	1-2	157 (16.2%)	164 (16.9%)	
	≥2	28 (2.9%)	38 (3.9%)	
Rural (*n* = 366)	<1	296 (80.8%)	288 (78.7%)	0.7574
	1-2	56 (15.3%)	63 (17.2%)	
	≥2	14 (3.8%)	15 (4.1%)	
**COVID-19 morbidity for the voivodeship**				
Morbidity < 40/ 100.000 (*n* = 413)	<1	328 (79.4%)	323 (78.2%)	0.8989
	1-2	69 (16.7%)	74 (17.9%)	
	≥2	16 (3.9%)	16 (3.9%)	
Morbidity 40-80/ 100.000 (*n* = 537)	<1	423 (78.7%)	415 (77.3%)	0.3318
	1-2	96 (17.8%)	93 (17.3%)	
	≥2	19 (3.5%)	29 (5.4%)	
Morbidity 80-160/ 100.000 (*n* = 209)	<1	173 (82.8%)	164 (78.5%)	0.4931
	1-2	30 (14.3%)	39 (18.6%)	
	≥2	6 (2.9%)	6 (2.9%)	
Morbidity >160/ 100.000 (*n* = 175)	<1	155 (88.6%)	152 (86.9%)	0.8781
	1-2	18 (10.3%)	21 (12.0%)	
	≥2	2 (1.1%)	2 (1.1%)	
Total (*n* = 1334)	<1	1079 (80.9%)	1054 (79.0%)	0.3657
	1-2	213 (15.9%)	227 (17.0%)	
	≥2	42 (3.2%)	53 (4.0%)	

Table S6. Analysis of declared fast food consumption in the studied group of Polish adolescents stratified by gender, age, size of the city and COVID-19 morbidity in voivodeship.

Category	Portions per week	Before COVID-19	During remote education due to COVID-19	p-Value
**Gender**				
Girls (n = 711)	<1	644 (90.6%)	637 (89.6%)	0.3381
	1-2	59 (8.3%)	59 (8.3%)	
	≥2	8 (1.1%)	15 (2.1%)	
Boys (n = 623)	<1	553 (88.8%)	550 (88.3%)	0.7835
	1-2	55 (8.8%)	54 (8.7%)	
	≥2	15 (2.4%)	19 (3.0%)	
**Age (years)**				
10-13 (n = 1017)	<1	929 (91.3%)	913 (89.8%)	0.2972
	1-2	75 (7.4%)	83 (8.2%)	
	≥2	13 (1.3%)	21 (2.0%)	
13-16 (n = 317)	<1	268 (84.5%	274 (86.4%)	0.4423
	1-2	39 (12.3%)	30 (9.5%)	
	≥2	10 (3.2%)	13 (4.1%)	
**Size of the city**				
Urban (n = 968)	<1	862 (89.0%)	850 (87.8%)	0.3647
	1-2	89 (9.2%)	92 (9.5%)	
	≥2	17 (1.8%)	26 (2.7%)	
Rural (n = 366)	<1	335 (91.5%)	337 (92.1%)	0.7263
	1-2	25 (6.9%)	21 (5.7%)	
	≥2	6 (1.6%)	8 (2.2%)	
**COVID-19 morbidity for the voivodeship**				
Morbidity < 40/ 100.000 (n = 413)	<1	362 (87.7%)	362 (87.7%)	0.5527
	1-2	45 (10.9%)	41 (9.9%)	
	≥2	6 (1.4%)	10 (2.4%)	
Morbidity 40-80/ 100.000 (n = 537)	<1	473 (88.1%)	470 (87.5%)	0.7585
	1-2	51 (9.5%)	50 (9.3%)	
	≥2	13 (2.4%)	17 (3.2%)	
Morbidity 80-160/ 100.000 (n = 209)	<1	196 (93.8%)	193 (92.4%)	0.6931
	1-2	11 (5.3%)	12 (5.7%)	
	≥2	2 (0.9%)	4 (1.9%)	
Morbidity >160/ 100.000 (n = 175)	<1	166 (94.5%)	162 (92.6%)	0.5919
	1-2	6 (3.4%)	10 (5.7%)	
	≥2	3 (1.7%)	3 (1.7%)	
Total (n = 1334)	<1	1197 (89.7%)	1187 (88.9%)	0.4135
	1-2	113 (8.5%)	113 (8.5%)	
	≥2	24 (1.8%)	34 (2.6%)	

Table S7. Analysis of declared frequency of eating their meals in front of the television in the studied group of Polish adolescents stratified by gender, age, size of the city and COVID-19 morbidity in voivodeship.

Category	Frequency	Before COVID-19	During remote education due to COVID-19	p-Value
**Gender**				
Girls (*n* = 711)	Never or rarely	271 (38.1%)	226 (31.8%)	0.0001
	1-2	146 (20.5%)	220 (30.9%)	
	≥3	204 (28.7%)	184 (25.9%)	
	7/week	90 (12.7%)	81 (11.4%)	
Boys (*n* = 623)	Never or rarely	205 (32.9%)	159 (25.6%)	<0.0001
	1-2	104 (16.7%)	182 (29.2%)	
	≥3	211 (33.9%)	192 (30.8%)	
	7/week	103 (16.5%)	90 (14.4%)	
**Age (years)**				
10-13 (*n* = 1017)	Never or rarely	383 (37.7%)	308 (30.3%)	<0.0001
	1-2	192 (18.9%)	319 (31.4%)	
	≥3	303 (29.8%)	271 (26.6%)	
	7/week	139 (13.6%)	119 (11.7%)	
13-16 (*n* = 317)	Never or rarely	93 (29.3%)	77 (24.3%)	0.1022
	1-2	58 (18.3%)	83 (26.2%)	
	≥3	112 (35.3%)	105 (33.1%)	
	7/week	54 (17.1%)	52 (16.4%)	
**Size of the city**				
Urban (*n* = 968)	Never or rarely	335 (34.6%)	269 (27.8%)	<0.0001
	1-2	172 (17.8%)	287 (29.7%)	
	≥3	310 (32.0%)	280 (28.9%)	
	7/week	151 (15.6%)	132 (13.6%)	
Rural (*n* = 366)	Never or rarely	141 (38.5%)	116 (31.7%)	0.0182
	1-2	78 (21.3%)	115 (31.4%)	
	≥3	105 (28.7%)	96 (26.2%)	
	7/week	42 (11.5%)	39 (10.7%)	
**COVID-19 morbidity for the voivodeship**				
Morbidity < 40/ 100.000 (*n* = 413)	Never or rarely	143 (34.6%)	127 (30.7%)	0.2042
	1-2	170 (41.2%)	199 (48.2%)	
	≥3	44 (10.6%)	34 (8.2%)	
	7/week	56 (13.6%)	53 (12.8%)	
Morbidity 40-80/ 100.000 (*n* = 537)	Never or rarely	188 (35.0%)	151 (28.1%)	0.0107
	1-2	207 (38.5%)	260 (48.4%)	
	≥3	56 (10.5%)	53 (9.9%)	
	7/week	86 (16.0%)	73 (13.6%)	
Morbidity 80-160/ 100.000 (*n* = 209)	Never or rarely	88 (42.1%)	67 (32.1%)	0.0117
	1-2	62 (29.7%)	94 (45.0%)	
	≥3	31 (14.8%)	22 (10.5%)	
	7/week	28 (13.4%)	26 (12.4%)	
Morbidity >160/ 100.000 (*n* = 175)	Never or rarely	56 (32.0%)	40 (22.8%)	0.0486
	1-2	70 (40.0%)	96 (54.9%)	

	≥3	25 (14.3%)	20 (11.4%)	
	7/week	24 (13.7%)	19 (10.9%)	
Total (n = 1334)	Never or rarely	475 (35.6%)	385 (28.9%)	<0.0001
	1-2	509 (38.2%)	649 (48.6%)	
	≥3	156 (11.7%)	129 (9.7%)	
	7/week	194 (14.5%)	171 (12.8%)	

Table S8. Analysis of declared number of days a week when they are physically active in the studied group of Polish adolescents stratified by gender, age, size of the city and COVID-19 morbidity in voivodeship.

Category	Frequency per week	Before COVID-19	During remote education due to COVID-19	p-Value
**Gender**				
Girls<br>(*n* = 711)	0-2/week<br>≥ 3/week	240 (33.8%)<br>471 (66.2%)	273 (38.4%)<br>438 (61.6%)	0.0772
Boys<br>(*n* = 623)	0-2/week<br>≥ 3/week	194 (31.1%)<br>429 (68.9%)	246 (39.5%)<br>377 (60.5%)	0.0025
**Age (years)**				
10-13<br>(*n* = 1017)	0-2/week<br>≥ 3/week	400 (39.3%)<br>617 (60.7%)	364 (35.8%)<br>653 (64.2%)	0.1090
13-16<br>(*n* = 317)	0-2/week<br>≥ 3/week	142 (44.8%)<br>175 (55.2%)	137 (43.2%)<br>180 (56.8%)	0.7489
**Size of the city**				
Urban<br>(*n* = 968)	0-2/week<br>≥ 3/week	401 (41.4%)<br>567 (58.6%)	368 (38.0%)<br>600 (62.0%)	0.1372
Rural<br>(*n* = 366)	0-2/week<br>≥ 3/week	141 (38.5%)<br>225 (61.5%)	133 (50.0%)<br>133 (50.0%)	0.0052
**COVID-19 morbidity for the voivodeship**				
Morbidity < 40/ 100.000<br>(*n* = 413)	0-2/week<br>≥ 3/week	170 (41.2%)<br>243 (58.8%)	150 (36.3%)<br>263 (63.7%)	0.1748
Morbidity 40-80/ 100.000<br>(*n* = 537)	0-2/week<br>≥ 3/week	221 (41.1%)<br>316 (58.9%)	204 (37.9%)<br>333 (62.1%)	0.3181
Morbidity 80-160/ 100.000<br>(*n* = 209)	0-2/week<br>≥ 3/week	83 (39.7%)<br>126 (60.3%)	76 (36.4%)<br>133 (63.6%)	0.5455
Morbidity >160/ 100.000<br>(*n* = 175)	0-2/week<br>≥ 3/week	68 (38.9%)<br>107 (61.1%)	71 (40.6%)<br>104 (59.4%)	0.8271
Total<br>(*n* = 1334)	0-2/week<br>≥ 3/week	542 (40.6%)<br>792 (59.4%)	501 (37.6%)<br>833 (62.4%)	0.1125

Table S9. Analysis of declared number of hours a day that they usually spend watching television in the studied group of Polish adolescents stratified by gender, age, size of the city and COVID-19 morbidity in voivodeship.

Category	Frequency per day	Before COVID-19	During remote education due to COVID-19	p-Value
**Gender**				
Girls (*n* = 711)	< 2 h/day	193 (27.1%)	106 (14.9%)	<0.0001
	≥ 2 h/day	518 (72.9%)	605 (85.1%)	
Boys (*n* = 623)	< 2 h/day	85 (13.6%)	45 (7.2%)	0.0003
	≥ 2 h/day	538 (86.4%)	578 (92.7%)	
**Age (years)**				
10-13 (*n* = 1017)	< 2 h/day	222 (21.8%)	56 (17.7%)	0.1299
	≥ 2 h/day	795 (78.2%)	261 (82.3%)	
13-16 (*n* = 317)	< 2 h/day	115 (11.3%)	36 (11.4%)	1.0000
	≥ 2 h/day	902 (88.7%)	281 (88.6%)	
**Size of the city**				
Urban (*n* = 968)	< 2 h/day	455 (47.0%)	272 (28.1%)	<0.0001
	≥ 2 h/day	513 (53.0%)	696 (71.9%)	
Rural (*n* = 366)	< 2 h/day	208 (56.8%)	138 (37.7%)	<0.0001
	≥ 2 h/day	158 (43.2%)	228 (62.3%)	
**COVID-19 morbidity for the voivodeship**				
Morbidity < 40/ 100.000 (*n* = 413)	< 2 h/day	83 (20.1%)	40 (9.7%)	<0.0001
	≥ 2 h/day	330 (79.9%)	373 (90.3%)	
Morbidity 40-80/ 100.000 (*n* = 537)	< 2 h/day	125 (23.2%)	70 (13.1%)	<0.0001
	≥ 2 h/day	109 (76.8%)	467 (86.9%)	
Morbidity 80-160/ 100.000 (*n* = 209)	< 2 h/day	57 (27.3%)	30 (14.3%)	0.0017
	≥ 2 h/day	152 (72.7%)	179 (85.7%)	
Morbidity >160/ 100.000 (*n* = 175)	< 2 h/day	24 (13.7%)	15 (8.6%)	0.1742
	≥ 2 h/day	151 (86.3%)	160 (91.4%)	
Total (*n* = 1334)	< 2 h/day	289 (21.7%)	155 (11.6%)	<0.0001
	≥ 2 h/day	1045 (78.3%)	1179 (88.4%)	
